# Categorisation of continuous exposure variables revisited. A response to the Hyperglycaemia and Adverse Pregnancy Outcome (HAPO) Study

**DOI:** 10.1186/1471-2288-10-103

**Published:** 2010-11-09

**Authors:** Kathrine F Frøslie, Jo Røislien, Petter Laake, Tore Henriksen, Elisabeth Qvigstad, Marit B Veierød

**Affiliations:** 1 National Resource Centre for Women's Health, Division of Obstetrics and Gynaecology, Oslo University hospital Rikshospitalet, Norway; 2 Department of Biostatistics, Institute of Basic Medical Sciences, University of Oslo, Norway; 3 Division of Obstetrics and Gynaecology, Oslo University Hospital Rikshospitalet, Norway; 4 Section of Endocrinology, Medical Department, Oslo University Hospital Rikshospitalet, Norway

## Abstract

**Background:**

Although the general statistical advice is to keep continuous exposure variables as continuous in statistical analyses, categorisation is still a common approach in medical research. In a recent paper from the Hyperglycaemia and Adverse Pregnancy Outcome (HAPO) Study, categorisation of body mass index (BMI) was used when analysing the effect of BMI on adverse pregnancy outcomes. The lowest category, labelled "underweight", was used as the reference category.

**Methods:**

The present paper gives a summary of reasons for categorisation and methodological drawbacks of this approach. We also discuss the choice of reference category and alternative analyses. We exemplify our arguments by a reanalysis of results from the HAPO paper.

**Results:**

Categorisation of continuous exposure data results in loss of power and other methodological challenges. An unfortunate choice of reference category can give additional lack of precision and obscure the interpretation of risk estimates. A highlighted odds ratio (OR) in the HAPO study is the OR for birth weight >90^th ^percentile for women in the highest compared to the lowest BMI category ("obese class III" versus "underweight"). This estimate was OR = 4.55 and OR = 3.52, with two different multiple logistic regression models. When using the "normal weight" category as the reference, our corresponding estimates were OR = 2.03 and OR = 1.62, respectively. Moreover, our choice of reference category also gave narrower confidence intervals.

**Summary:**

Due to several methodological drawbacks, categorisation should be avoided. Modern statistical analyses should be used to analyse continuous exposure data, and to explore non-linear relations. If continuous data are categorised, special attention must be given to the choice of reference category.

## Background

Although the general statistical advice is to keep continuous exposure variables as continuous in statistical analyses [1,2], categorisation of continuous variables is still a common approach in medical research [3,4]. In the present paper we revisit this issue, and we have chosen to exemplify our arguments through a discussion and reanalysis of data from the recently published paper "Hyperglycaemia and Adverse Pregnancy Outcome (HAPO) Study: associations with maternal body mass index." [5].

The main objective of the international, multicentre HAPO study was to clarify the risk of adverse outcomes associated with various degrees of glucose intolerance during pregnancy, including degrees less than overt diabetes [6]. With its impressive sample size, double-blinding of glucose levels and thoroughly developed standardized routines for data collection, this is already a well-cited and highly valued study in the fields of obstetrics and endocrinology. In total, 25 505 women were enrolled in the study from 2000 to 2006. Glucose tolerance was measured by a 75-g 2-h oral glucose tolerance test (OGTT) in a large, heterogeneous, multinational, ethnically diverse cohort of women at approximately 28 weeks of gestation. The participating women, caregivers and HAPO staff were blinded to glucose tolerance values, except when predefined thresholds were met. Height and weight were also measured by a standardized procedure at the OGTT visit. Both glucose tolerance and body mass index (BMI, kg/m^2^) were measured on continuous scales. In the study protocol it is argued for a possible continuous relation between glucose and pregnancy outcomes [6]. However, in the statistical analyses, BMI was categorised.

Our aim is to discuss arguments for categorisation of exposure variables and the corresponding methodological challenges of doing so. We also discuss issues that need attention if categorisation is the strategy of choice. Furthermore, we show how different choices of reference category can influence the results presented in the HAPO paper. Finally, we present alternative strategies to that of categorisation of continuous variables.

### Regression analysis and linearity assumptions

The primary analytic tool in the HAPO paper [5] was logistic regression analysis. In standard logistic regression analyses, linearity in the logit is assumed [7]. Similarly, in linear regression, a linear relation is assumed between the exposure variable and the response variable. These assumptions give easily interpretable results for continuous exposure variables. However, a linearity check should always be carried out. If the observed data fulfil the linearity assumptions, the general advice is to keep continuous variables as continuous in such analyses [1,2]. A wide range of linearity diagnostics is available for different regression models [7]. Although we will use logistic regression analyses from the HAPO paper as our example, our arguments apply to other regression models as well. The authors of the HAPO paper have discarded a linear model with BMI as a continuous exposure variable because of a statistically significant squared term of BMI. This is not in itself a valid argument for rejection of a linear model, unless additional linearity diagnostics have also been used. In any case, presence of non-linearity must be reflected in regression models. Several approaches are possible. The most commonly used approach, also taken in the HAPO paper, is to categorise the data [3,4].

### The cost of categorisation of continuous data

Categorisation of continuous exposure data is intriguing because it seems to represent a simple solution to a methodological challenge. It is a common belief that categorical exposure data are both more robust, i.e. not easily affected by small departures from model assumptions, easier to interpret and more feasible to present than their continuous counterparts. Categorisation also mirrors clinical practice. Research results are crucial in the development of clinical medical practice where decisions typical are categorical in nature. The similarities between clinical decision-making and a categorical data analysis and presentation approach can make reading and understanding scientific papers with categorical data easier for clinicians. However, categorisation comes with some costs. Firstly, there is a loss of power due to reduced variability in the data. Several papers have addressed this issue and simulation studies have been used to quantify the loss [1,2]. In a study as large as the HAPO study, there is enough information to retain sufficient power even after the categorising of data. Indeed, the sample size calculations for the HAPO study were based on categorised data [6]. Paradoxically, one consequence of large sample sizes is that even small deviations from linearity can be detected, which again leads to categorisation of the originally continuous variables, resulting in loss of power and the need for larger sample sizes. Secondly, categorisation conceals information about the details of the non-linear relation. When categorising, one assumes that the relation between the independent and the dependent variable is constant within intervals. This implies that any change in effect within an interval will be lost, also biologically plausible ones. Less obvious, but also important consequences of categorisation are those related to residual confounding [1,2], increased risk of false positive results [1,2] and issues about misclassification [8], which all may give unintentionally biased results. It has been shown that less of the confounding will be removed if one adjusts for a categorised variable, instead of the original continuous variable [1,2]. Further, choice of cut-off values can significantly impact effect measures, e.g. odd ratios, and can thereby lead to artificial associations between variables [1,2]. Finally, most exposure variables are prone to measurement errors. When categorising a continuous variable, nondifferential errors can lead to differential misclassification [8].

### Categorisation in the HAPO paper

Assuming that the choice of categorising data is made despite of the shortcomings of such a strategy, cut-off values and reference category must be chosen with care. It is common to use internationally and clinically accepted categories, if such exist. The HAPO paper [5] focuses on BMI as the exposure variable for several pregnancy outcomes. The international classification of BMI (kg/m^2^) in adults suggested by WHO [9] is "underweight" (<18.5), "normal weight" (18.5-24.9), "overweight" (25.0-29.9) and "obese class I-III" (30.0-34.9, 35.0-39.9 and ≥40, respectively). In the HAPO study, BMI was measured only at gestational weeks 24-32 (at the OGTT). Due to the expected weight gain during the first 24-32 weeks of the pregnancy the WHO categories did not readily apply. Instead, "comparable category limits for BMI" at 28 weeks of gestation were estimated by a regression analysis of BMI measured at the OGTT on recalled pre-pregnancy BMI and gestational age at the OGTT: <22.6, 22.6-28.4, 28.5-32.9, 33.0-37.4, 37.4-41.9 and ≥42.0 kg/m^2^.

This usage of regression modelling deserves a remark. A regression model consists of two main elements; a deterministic term, linking the exposure to the outcome, and an error term which incorporates the residual variance of the observations, which is not expressed by the link function. This implies that one cannot merely transform the mid-pregnancy BMI to a predicted pre-pregnancy BMI, based on the regression estimates, as part of the variance has been discarded when the pre-pregnant BMI was estimated. Additionally, it is not easy to deduct how this could influence misclassification issues.

In statistical analysis of categorical data, the reference category is the category against which the others are compared [7]. Both the biological interpretation of the estimated association and the number of observations should be considered when choosing the reference category. In the HAPO paper, the estimated "normal range" BMI category at gestational week 24-32 was 22.6-28.4 kg/m^2^. With its almost 12 000 (51%) women it was also the largest group, making it the preferred reference category both by means of a natural reference and by considerations of statistical power. However, the lowest BMI group, labelled "underweight", with 2989 (13%) women was chosen as the reference. This resulted in broader confidence intervals, and somewhat misleading effect estimates. Clinically, the underweight group is susceptible to having additional health problems, like eating disorders or chronic diseases influencing body weight. Thus, there are several reasons why using the "underweight" group as the reference could influence the estimated associations in the HAPO study.

The HAPO paper estimated associations between BMI and 11 different outcomes. The results were presented as frequencies and adjusted effect estimates (odds ratios, ORs, and 95% confidence intervals, CIs) from two different multiple logistic regression models. The crude ORs were not given, but can easily be calculated from the frequencies in the tables. We have focused on the macrosomia (high birth weight) results, and Table 1 shows results from logistic regression analyses of the effect of BMI on macrosomia, with two different choices of reference category. We first considered the crude results with reference categories BMI <22.6 kg/m^2 ^or BMI 22.6-28.4 kg/m^2^. Although the same increasing trend is seen, the two analyses give different impressions to the reader. By using "underweight" as the reference group, the increasing trend is striking, with a nearly five-fold increased risk for macrosomia in the highest BMI group ("obese class III"). When "normal range" is used as the reference group, the similarities of the three highest BMI groups are clearly seen, in addition to the statistically significantly lower OR for the "underweight" group. The lack of increase in ORs for the three highest BMI groups is even more convincing for the adjusted ORs. In addition, the table shows the standard errors of the betas in the logistic regressions and the widths of the unadjusted 95% CIs, illustrating the loss in statistical power by using a small group as the reference, compared to using the larger normal range group as the reference. It should be noted that using SE for the betas instead of CI for the ORs as measures of precision of the estimates show much less exaggeration of lack of precision when changing reference category. However, most scientific papers that present results from logistic regression focus on ORs rather than betas. We have therefore chosen to present both, also the widths of the CIs. By changing the reference category, the main conclusions in the paper would not be altered, but the effect sizes would be relative to pregnant women with a normal range BMI, as well as being more precisely estimated.

**Table 1:** The effect of BMI on macrosomia: Different reference categories influence effect estimates and precision.

		Crude analysis						Adjusted analysis**			
								HAPO model I		HAPO model II	
**Reference category**		**BMI <22.6**			**BMI 22.6-28.4**			**BMI <22.6**	**BMI 22.6-28.4**	**BMI <22.6**	**BMI 22.6-28.4**

**BMI (kg/m**^**2**^)*	**n**	**Beta (SE)**	**OR (95% CI)**	**Width of CI**	**Beta (SE)**	**OR (95% CI)**	**Width of CI**	**OR**	**OR**	**OR**	**OR**

<22.6	2974	0	1.00		-0.80 (0.10)	0.45 (0.37-0.55)	0.2	1.00	0.45	1.00	0.46
22.6-28.4	11934	0.80 (0.10)	2.23 (1.83, 2.71)	0.9	0	1.00		2.24	1.00	2.17	1.00
28.5-32.9	5127	1.27 (0.10)	3.57 (2.91, 4.38)	1.5	0.47 (0.05)	1.60 (1.44-1.78)	0.3	3.62	1.62	3.31	1.52
33.0-37.4	2064	1.47 (0.11)	4.36 (3.49, 5.45)	2.0	0.67 (0.07)	1.96 (1.71-2.25)	0.5	4.43	1.98	3.89	1.79
37.5-41.9	735	1.50 (0.14)	4.47 (3.40, 5.88)	2.5	0.70 (0.11)	2.01 (1.63-2.48)	0.9	4.52	2.02	3.80	1.76
≥42.0	383	1.55 (0.17)	4.71 (3.38, 6.56)	3.2	0.75 (0.14)	2.12 (1.60-2.80)	1.2	4.55	2.03	3.52	1.62

* BMI categories are based on BMI measured at 28 weeks gestation, and the categories correspond to pre-gestational BMI categories as suggested by WHO: "underweight" (<18.5), "normal weight" (18.5-24.9), "overweight" (25.0-29.9) and "obese class I-III" (30.0-34.9, 35.0-39.9 and ≥40, respectively) [4].

** HAPO Model I: Adjusted for the variables used in estimating 90th birth weight percentiles (gender, gestational age, ethnicity, field centre, and maternal parity), age, height and gestational age at the oral glucose tolerance test, smoking, alcohol use, hospitalisation before delivery and any family history of diabetes. Model II: Model I with additional adjustment for fasting plasma glucose and mean arterial pressure [4].

The table shows results from logistic regression analyses of the effect of BMI on macrosomia, with two different choices of reference category. All results are based on the HAPO paper [4].

### Alternatives to categorisation

Application of more complex modern statistical techniques is necessary if we want to keep variables continuous in situations where non-linearity is evident. Suitable statistical methods include piecewise linear regression [10] and spline based techniques [11], for example generalised additive models (GAMs). By applying such techniques, one will gain statistical power, avoid unintended methodological challenges based on classification problems, as well as allowing the data to provide more explicit details about the non-linear relations. This could include apparent change-points or thresholds values where a change in the outcome variable can be observed, piecewise linear relations, "plateau" effects and so on. The existence and locations of change-points can further be formally tested for and estimated [10]. In the HAPO paper, a plateau effect for the higher BMI categories is discussed. Both clinical practice and physiological theories would benefit from a statistical test for the existence of a plateau effect, and an estimate of the BMI value where the risk levelled off. Application of more complex analytical tools will to some extent complicate the technical analysis and the task of applying and communicating the research results. However, this should not prevent a more optimal analysis.

## Summary

Applying and communicating results from statistical analyses is a challenging aspect of the scientific process. The results should be presented in a simple, yet balanced manner, and should be based on adequate statistical methods. Researchers should be aware of the trade-off between simplicity in presentation and unintended methodological challenges arising from simplifications. Particularly, if one chooses to categorise continuous exposure variables, special attention must be given both to the definition of categories and the choice of reference category. If we apply modern statistical analysis techniques, we might extract more detailed knowledge and get a better utilisation of our data.

## Abbreviations

HAPO Study: Hyperglycaemia and Adverse Pregnancy Outcome Study; BMI: Body Mass Index defined as weight/height^2^, where weight is measured in kilograms (kg) and height is measured in meters (m); OGTT: Oral glucose tolerance test; GAMs: Generalised additive models.

## Competing interests

There were no competing interests, and no personal relationships, academic competition, or intellectual commitments that might bias the work or interfere with objective judgment.

## Authors' contributions

KFF did the statistical analyses. All authors contributed to the discussions about the topic, revision the manuscript and to the final approval of the manuscript.

## Pre-publication history

The pre-publication history for this paper can be accessed here:

http://www.biomedcentral.com/1471-2288/10/103/prepub
